# Notice

**Published:** 1892-09

**Authors:** 


NOTICE.—No contracts for advertisements will be accepted unless signed by D. H. Howell, Business Manager.

